# Determination of Proton Relaxivities of Mn(II), Cu(II) and Cr(III) added to Solutions of Serum Proteins

**DOI:** 10.3390/molecules14041537

**Published:** 2009-04-14

**Authors:** Mehmet Zafer Köylü, Sezai Asubay, Ali Yilmaz

**Affiliations:** Department of Physics, University of Dicle, 21280 Diyarbakir, Turkey

**Keywords:** MRI, T_1_, T_2_, Mn(II), Cu(II), Cr(III), Serum proteins

## Abstract

Relaxometric studies are still of scientific interest due to their use in medicine and biology. In this study, proton T_1_ and T_2_ relaxivities of Mn(II), Cu(II) and Cr(III) in water were determined in the presence and absence of various proteins (albumin, α-globulin, γ-globulin, lysozyme, fibrinogen). The 1/T_1_ and 1/T_2_ in all solutions are linearly proportional to the concentration of the paramagnetic ions. Mn(II) has the great influence to alter relaxations in all protein solutions, while Cu(II) and Cr(III) have a poor influence on the relaxations. In addition, Mn(II) and Cu(II) are bound to each protein, but Cr(III) is not bound to any protein.

## Introduction

Paramagnetic ions have continuously been in the center of NMR studies due to their large magnetic dipole moments [1,2,3,4,5,6,7,8,9,10,11,12]. *In vitro* relaxation studies on paramagnetic ion-protein interactions provide useful information about binding of an ion to protein, and also about the environments of the ion binding sites [2,13]. Such studies also provide a background for developing contrast agents (naked ion chelates) used in Magnetic Resonance Imaging (MRI) for diagnostic purposes [14,15,16,17,18]. On the other hand, the relaxivity of an ion is defined as the relaxation rate increase per unit concentration of ion. Relaxometric studies give information about the effectiveness of the ions to alter relaxation [3,4,14,19,20]. For these reasons, such studies may be useful for selecting the appropriate paramagnetic ions for studies on ion-protein interactions. Studies on the relaxivity of the naked state of the ions may also provide a background for development of further chelates of these ions specific to serum proteins. 

Albumin, α-globulin, γ-globulin, lysozyme and fibrinogen are the main serum proteins present in circulating blood. Serum proteins are the native carriers of some paramagnetic ions such as iron and copper. They are also the carriers of drugs and contrast agents to target organs [14,21]. For these reasons, these proteins were selected for this study. On the other hand, relaxometric studies on naked transient ions have already been made for some biological fluids and blood [4,14,19,20,22]. Relaxation studies of chelates of these ions have also been carried out [14,15,16,17,18]. To the best knowledge of the authors, except for albumin solutions and manganese ions, no such study has been done for a specific serum protein containing the naked state of the ions. Therefore, a new study on the relaxivity of some transition metal ions in protein solutions should give further information for this field.

In this study, The T_1_ and T_2_ relaxation times of different protein solutions (Albumin, α-globulin, γ-globulin, lysozyme, fibrinogen) were measured versus paramagnetic ions such as Mn(II), Cu(II) and Cr(III). Similar experiments were repeated in water for comparison. The data was used to determine effectiveness of each ion to alter relaxation. It was also utilized to determine whether an ion is bound or not.

## Results and Discussion

**Figure 1:**
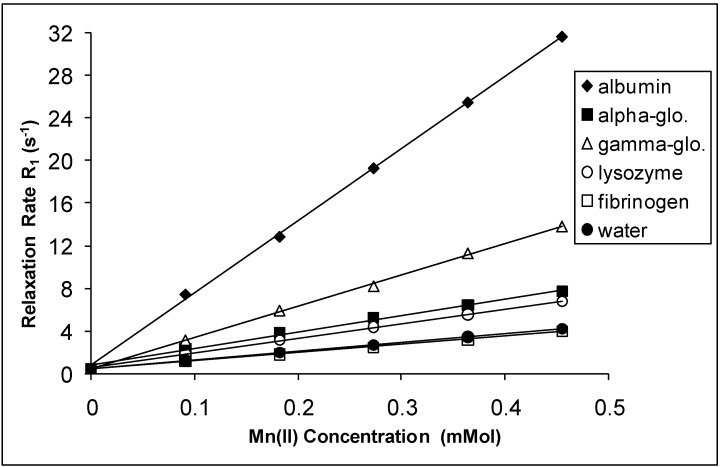
The Spin-lattice Relaxation rates (R_1_) of protein solutions versus concentration of Mn(II).

**Figure 2:**
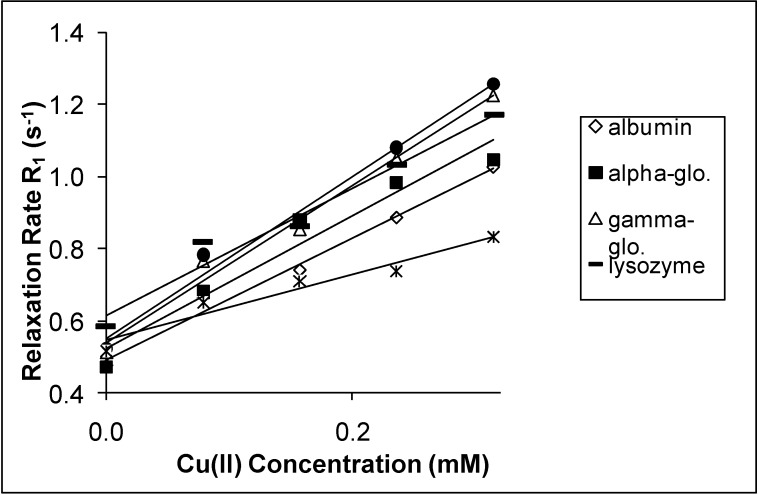
The Spin-lattice Relaxation rates (R_1_) of protein solutions versus concentration of Cu(II).

**Figure 3:**
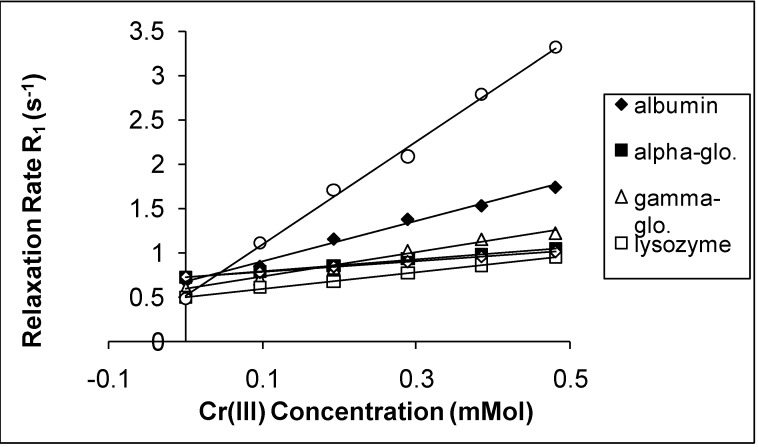
The Spin-lattice Relaxation rates (R_1_) of protein solutions versus concentration of Cr(III).

**Figure 4:**
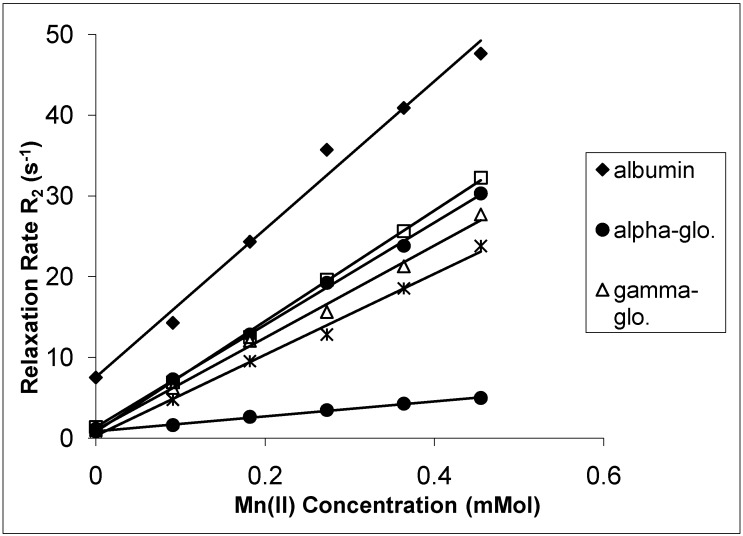
The Spin-Spin Relaxation rates (R_2_) of protein solutions versus concentration of Mn(II).

**Figure 5:**
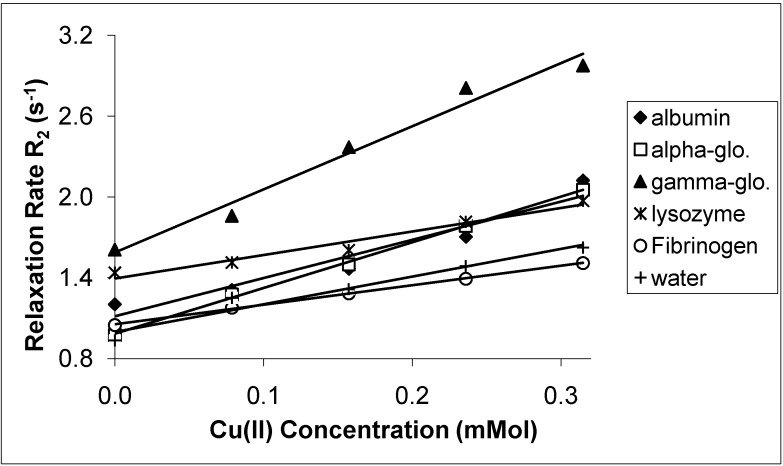
The Spin-Spin Relaxation rates (R_2_) of protein solutions versus concentration of Cu(II).

**Figure 6:**
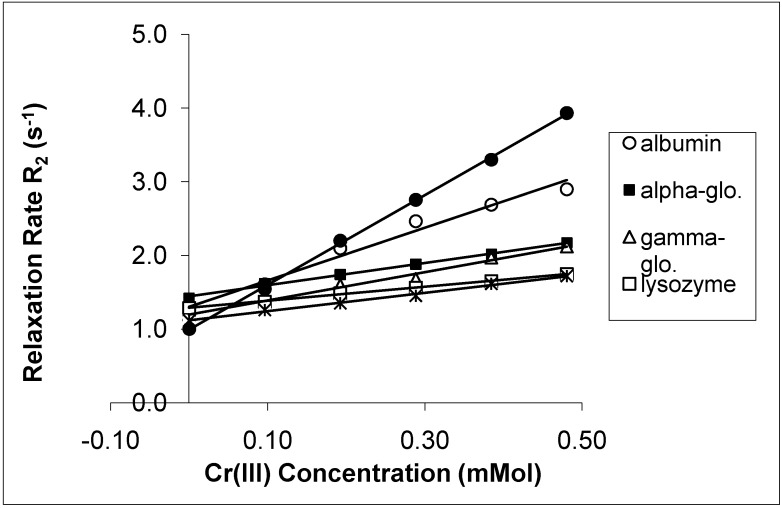
The Spin-Spin Relaxation rates (R_2_) of protein solutions versus concentration of Cr(III).

The spin-lattice relaxation rates (1/T_1_ or R_1_) in the solutions of the proteins containing Mn(II), Cu(II) and Cr(III) are shown in Figure 1, Figure 2 and Figure 3, respectively, while their respective spin-spin relaxation rates (1/T_2_or R_2_) are shown in Figure 4, Figure 5 and Figure 6. 

It is seen that the relaxation rates in all protein solutions increase linearly with the ion concentrations. A high correlation between the relaxation rates and the ion concentration was found for each fit. This is completely consistent with previous works, indicating a linear relation between relaxation rates and concentrations [4,8,14,19,20]. On the other hand, the observed relaxation rates can be expressed as follows:

*R_i_* = *R*_*i*0_ + *r_i_C* (i: 1 and 2) (1)

where 1 and 2 denote T_1_ and T_2_ relaxations, respectively [14]. The R_i_ and R_i0_ are the relaxation rates of protein solutions in the presence and absence of paramagnetic ions. The slope of the relation is equal to the relaxation rate increase per unit concentration of added ion. Accordingly, the slopes are the relaxivities of the added ions which are denominated as r_1_ for T_1_ and as r_2 _for T_2_ [4,14,19,20]. The relaxivities (r_1_ and r_2_) of the ions in the protein solutions are shown in Table 1 and Table 2.

**Table 1:** The r_1_ values of the paramagnetic ions in solutions of albumin, α-globulin, γ-globulin, lysozyme, fibrinogen and water.

Ions	Albumin	α-globulin	γ-globulin	Lysozyme	Fibrinogen	Water
Mn(II)	67,91	29,40	15,61	13,73	7,66	8,14
Cu(II)	1,68	1,84	2,18	1,76	2,24	0,90
Cr(III)	2,27	0,68	1,38	0,93	0,61	5.81

**Table 2:** The r_2_ values of the paramagnetic ions in solutions of albumin, α-globulin, γ-globulin, lysozyme, fibrinogen and water.

Ions	Albumin	α-globulin	γ-globulin	Lysozyme	Fibrinogen	Water
Mn(II)	91,56	57,05	63,31	68,40	50,07	9,28
Cu(II)	2,84	4,68	3,39	1,75	1,45	2,05
Cr(III)	3,58	1,51	1,92	0,95	1,24	6,08

Both r_1_ and r_2_ of the Mn(II) are higher than those of the other ions for all protein solutions. All the r_2_ relativities of the naked Mn(II) are higher than r_1_ for all solutions. The data indicates that Mn(II) is the most efficient relaxer for the solution of each protein. This is in very good agreement with the results of other fluids containing manganese ions [4,14,19,20]. The relaxivities of Cu(II) and Cr(III) ions are relatively poor. The results of Cu(II) and Cr(III) are consistent with earlier findings in other fluids [1,8,19,20]. 

In the protein solutions containing added ions, some added ions are free, but some are bound to proteins [2,13]. Then the free and bound hydration spheres occur in the solutions as a result of both states of the ions. There is also a fast chemical exchange of water between free and bound spheres [14]. This is the cause of the linear relationship between the relaxation rates and the ion concentrations. This is also the cause of measuring the relaxivity of the ions from slopes of the figures given above. On the other hand, the relaxation rates of water in hydration sphere of free ions are caused by dipolar interaction between water protons and the paramagnetic ion [4,14,19,20]. The contact interaction may also contribute to the relaxation mechanism [23]. The simplified Solomon-Bloembergen equations regarding the relaxation of free spheres are as follows [23]:

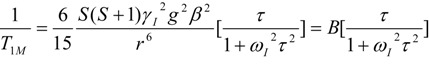
(2a)

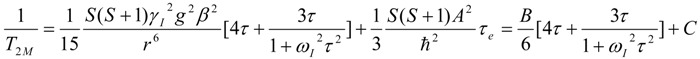
(2b)
where *B* denotes 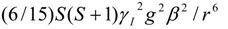 and the second term in Eq. 2b or C represents the contact interaction. The term given in Eq. 2a and the first term in Eq. 2b arise from the dipole-dipole interaction between the electron spin *S* and the nuclear spin *I*, characterized by an effective correlation time *τ*, and the second term in Eq. 2b arises from modulation of the contact interaction characterized by a correlation time *τ_e_* and a coupling constant *A* [23]. *ω_I_* and *r* are the nuclear Larmor precession frequency and the ion-proton interatomic distance, respectively. The constants *γ_I_*, *g*, *β* and *ħ* follow the usual notation. Both dipolar and scalar interactions may be modulated by the rotation of aquo-complex (*τ_r_*), life time of water molecule on the complex (*τ_m_*), longitudinal electron spin relaxation (*τ_s_* = *T*_1_*_E_*) and transverse electronic relaxation time *T*_2_*_E_*. The Eq. 2 is also valid for bound spheres. However, the correlation times are significantly changed upon binding of the ion to protein. This is the source of the increased relaxivity or enhanced relaxation in protein solutions containing added ion. Eq. 2 was particularly written for Manganese ions [23]. According to Eq. 2, T_1_ is caused by dipolar interactions, but T_2_ takes a contribution from contact interaction, too. The binding of an ion to protein can be evaluated from the following equation [24,25]:

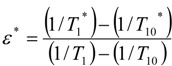
(3)
where ε^*^ is the relaxation rate enhancement caused by the ions. T_1_^*^ is the observed relaxation time in the presence of proteins and ions, whereas T_10_^*^ is the observed relaxation time of similar solution in the absence of ions. T_1_ is the observed relaxation rate of water containing ion but, T_10_ is the observed relaxation rate of the pure water.

**Table 3:** The proton relaxation rate enhancements (ε^*^) calculated from Equation 3.

	Mn(II)	Cu(II)	Cr(II)
albumin	8.6	1.6	0.4
α-globulin	3.6	2.0	0.1
γ-globulin	2.2	2.1	0.2
lysozyme	1.8	1.8	0.2
fibrinogen	0.9	2.2	0.1

It is known from literature that ε^*^ > 1 is an indicator for the binding of an ion to protein [24]. Then Table 3 shows that Mn(II) and Cu(II) ions are bound to proteins but Cr(III) is not. However, ε^*^ values for Mn(II) in fibrinogen solution indicates that Mn(II) is not bound to fibrinogen. Despite the findings of Tablo 3, the binding of the current ions to the proteins under consideration should be checked further by taking their precise concentrations and pH of solutions into account.

## Conclusions

The 1/T_1_ and 1/T_2_ increase linearly with ion concentrations. Mn(II) has a high influence to alter relaxation of each protein solution studied. Data show that Mn(II) and Cu(II) are complexed with all proteins. The data also suggest that Cr(III) is not bound to the proteins.

## Experimental

### Samples

The proteins (albumin, α-globulin, γ-globulin, lysozyme and fibrinogen) were purchased from Sigma. The chloric salts of the paramagnetic ions [MnCl_2_·2H_2_O, CuCl_2_·2H_2_O and CrCl_2_·6H_2_O] were used for the experiments. Aqueous solutions of 2.5g/100 mL of each of albumin, α-globulin, γ-globulin, lysozyme and 1g/100 mL of fibrinogen were prepared. The relaxation measurements were made against stepwise addition of each paramagnetic ion to the protein solutions. The concentrations of added ions in each set were altered from 0 to 25 μg/mL in increments of 5 μg/mL of each of Mn(II), Cu(II) and Cr(III) ions. The samples were transferred into cylindrical glass tubes which are 1.2 cm in diameter and 10 cm in height and were placed in a plastic phantom. Five protein solution phantoms with added ions (each containing one type of protein) and one water solution phantom containing doped ions were prepared. The pH of samples was between 7.4-7.7.

### Measurements

T_1_ and T_2_ measurements were made by 1.5T MRI (Philips Medical Systems, Intera, Netherlands) at a room temperature of 22 ^o^C, using a head coil. The field of view was 150 x 150 mm and the acquisition matrix was 256 x 256. The coronal images of a slice 14 mm thick was reconstructed by a 2D-FT technique. Then T_1_ measurements were performed by using a two-point ratio method with a mixed sequence where an inversion recovery (IR) and a spin echo (SE) are consecutively combined in one cycle. SE and IR repetition times, T_R_, were chosen to be 5,000 ms and 6,000 ms to allow a full recovery of magnetization, whereas the delay time T_1_ between the 180^o^ and 90^o^ pulses of the IR part of the mixed sequence was 800 ms. T_2_ measurements were carried out by the Carr-Purcell Meiboom-Gill pulse sequence with eight echoes. T_R_ was set at 5000 ms and echo delays were increased stepwise from 20 to 160 ms in increments of 20 ms. The standard deviations derived by the system for values of T_1_ and T_2_ of individual samples in the phantom ranged from 1% to 2 %. 
